# HMGA1 As a Potential Prognostic and Therapeutic Biomarker in Breast Cancer

**DOI:** 10.1155/2022/7466555

**Published:** 2022-11-26

**Authors:** Tian Wang, Ting Zhou, Fangfang Fu, Yingyan Han, Yan Li, Ming Yuan

**Affiliations:** Cancer Biology Research Center (Key Laboratory of the Ministry of Education), Tongji Hospital, Tongji Medical College, Huazhong University of Science and Technology, Wuhan, China

## Abstract

**Background:**

High-mobility group AT-hook1 (HMGA1) protein plays an important role in various diseases. However, the contribution of HMGA1 in breast cancer remains to be tapped.

**Methods:**

The expression of HMGA1 was analyzed in The Cancer Genome Atlas (TCGA) and TIMER database, and immunohistochemistry was performed in 39 breast cancer (BC) patients. The correlation between HMGA1 expression and prognosis was evaluated using Kaplan–Meier plotter (KM plotter) in patients with breast cancer. Then, cBioPortal and bc-GenExMiner were requisitioned to analyze the contribution of HMGA1 expression to clinical features. In order to reveal the function of HMGA1 in breast cancer cells, enrichment analysis was performed using the clusterProfiler R software package. Moreover, CCK8 assay, EdU assay, and Cell Cycle Assay were performed to assess the proliferation, and transwell assay was used to evaluate cell migration and invasion. Flow cytometry was used to explore the role of HMGA1 on cell apoptosis. After that, the effect of HMGA1 on signaling pathways in BC cells was detected by western blot.

**Results:**

HMGA1 was highly expressed in a variety of tumors tissues, including BC. High HMGA1 expression was correlated with poor prognosis in BC patients. Meanwhile, HMGA1 expression was increased in molecular phenotypes with poor prognosis (ER-, PR-, and HER2+) and associated with high-grade group, lymph node metastasis, and NPI (Nottingham Prognostic Index). Further, function analysis revealed HMGA1 was enriched in DNA replication and cell cycle pathways in breast cancer. Moreover, knockdown of HMGA1 caused apoptosis, inhibited proliferation, migration, and invasion of MCF-7 and MDA-MB-231 cells, in which the oncogenic signaling pathway of PI3K/AKT/MMP9 played a critical role.

**Conclusions:**

HMGA1 was important for breast cancer progression and was a critical prognostic indicator, prompting a potential therapeutic target of breast cancer.

## 1. Introduction

Breast cancer with an incidence of more than 10% is considered the most common female malignancy in the world [1]. Despite rapid advances in diagnosis and treatment, the prognosis of many breast cancer patients remains poor, and mortality of breast cancer remains a major challenge [2]. Metastasis is mainly responsible for treatment failure and 90% of cancer-related deaths in breast cancer [3]. Approximately 20%-30% early breast cancer patients will experience distant metastatic relapse. The 5-year overall survival rate for breast cancer patients without metastasis is greater than 80%, while distant metastasis can lead to a significant reduction to about 25% [4]. However, the regulatory mechanisms that conduce to cancer metastasis in breast cancer have not been expounded. Therefore, it is urgent to identify powerful prognostic predictors and novel therapeutic approaches for treatment of breast cancer.

High-mobility group AT-hook1 (HMGA1), located at chromosomal locus 6p21, is a protein that plays an important role in the assembly of enhancers of transcription factors and cofactors [5]. HMGA1 proteins are abundant during embryogenesis and tumorigenesis and are reported as a key molecule inducing transcriptional networks involved in self-renewal and pluripotency in embryonic stem cells [6]. With abnormally expression in most aggressive tumors, high expression of HMGA1 portends poor differentiation of tumor cells and adverse clinical outcomes of patients [7–9]. Further, accumulating researches have indicated that HMGA1 is critical for the progression of malignant tumors, including ovarian cancer, cervical cancer, and lung cancer [10–12], accelerating the malignant progression of tumors through influencing DNA replication, cell proliferation, and epithelial-to-mesenchymal transition (EMT) [5, 13]. However, the prognostic value of HMGA1 in breast cancer tissues and the mechanism in tumor progression remain to be determined.

In this study, the role of HMGA1 was evaluated in different types of tumors in The Cancer Genome Atlas (TCGA). We further investigated the association of HMGA1 expression with distinct receptors and molecular pathways in breast cancer. Finally, we explored the role of HMGA1 silencing on proliferation, apoptosis, migration, and invasion in breast cancer cells. These findings advance the understanding of HMGA1 and indicate that HMGA1 is a promising biomarker for breast cancer diagnosis and prognosis.

## 2. Materials and Methods

### 2.1. HMGA1 Gene Expression Analysis

The mRNA expression of HMGA1 in pan-cancer data was analyzed by using the Tumor Immune Estimate Resource (TIMER) database (https://cistrome. shinyapps.io/timer/). RNA-seq data of BC patients were downloaded from TCGA (https://www.cancer.gov/).

### 2.2. Survival Analysis

Survival analysis of breast cancer patients was performed in KM Plotter (http://www.kmplot.com) and bc-GenExMiner (http://http://bcgenex.centregauducheau.fr/), which was determined based on the hazard ratios (HRs) and log-rank *P* value. To distinguish between high and low expression of HMGA1, we used the median as the cut-off point for survival analysis, which is the number in the middle of a sequential set of data.

### 2.3. Correlation Analysis of HMGA1 and Clinicopathological Features

Online databases that contained cBioportal (http://www.cbioportal.org) and bc-GenExMiner were used to analyze the correlation between the HMGA1 expression and clinicopathological features.

### 2.4. Function Analysis

Pearson correlation analysis of HMGA1 mRNA and other mRNAs in breast cancer was performed using TCGA data. GSEA analysis was performed using the clusterProfiler package in R. GO analysis was performed using the EnrichGO function in the clusterProfiler package in R.

### 2.5. Cell Culture and Transfection

MDA-MB-231 cells were grown in L-15 medium containing 10% FBS at 37°C with 5% CO_2_. MCF-7 was cultured in Dulbecco's modified Eagle's medium (DMEM, Gibco) containing 10% fetal bovine serum (FBS, Gibco). The small interfering RNA (siRNA) was provided by RIBOBIO (Guangdong, China). The negative control RNA duplex (NC) was nonhomologous to any human genome sequences. We have designed three siRNA sequences and tested the targeted effect in breast cancer cells and finally chose siHMGA1-3 (Supplementary Figure 1). The sequence of HMGA1 siRNA-3 was 5′-AGCGAAGTGCCAACACCTA-3′. All transfections were performed with Lipofectamine® 3000 Transfection Reagent (Invitrogen) according to the manufacturer's protocol.

### 2.6. Transwell Assay

Migration assays were performed using Matrigel-free transwell chambers (Coring, USA), and invasion assays were carried out with Matrigel (BD Biosciences). Cells were seeded into the upper chamber of the transwell chamber (BD Biosciences, Franklin Lake, NJ) with a density of 2 × 10^5^ cells/ml. After 48 h at 37°C, cells on the top of the incubator were removed. Cells on the bottom were fixed with 4% paraformaldehyde for 10 min, and then cells stained with crystal violet (Beyotime, China) for 5 min and counted in microscope.

### 2.7. Cell Counting Kit (CCK8) Assay

Cells with a density of 5000 cells per well were seeded into 96-well plates. After incubated with 10 *μ*l CCK8 reagents, cells were detected in 24 h, 48 h, and 72 h using microplate reader (Molecular Devices, Rockford, IL, USA) with optical density value at 460 nm.

### 2.8. EdU Assay

We assessed cell proliferation using 5-ethynyl-2′-deoxyuridine (EdU) according to the manufacturer's instructions (BeyoClick™ EdU cell Proliferation Kit with Alexa Fluor 555). Briefly, cells (2 × 10^4^/well) were cultured in 8-well slide and incubated with 50 umol/L EdU (1 : 1000) for 12 hours. Cells were fixed with 4% formaldehyde at 37°C for 20 minutes and soaked in 0.5% Triton X-100 at 37°C.Then, add 100 *μ*l of Apollo reaction cocktail and incubate in the shade for 30 minutes. After several washes with PBS, the nuclei were stained with 4′,6′-diamidino-2-phenylindole (DAPI) at 37°C for 20 minutes. The EdU-labelled cells were observed by laser scanning confocal microscopy (Leica SP8) and normalized to the total number of cells stained with DAPI.

### 2.9. Flow Cytometry

For analysis of cell cycle assay, treated cells were collected, fixed overnight in 70% ethanol at 4°C, washed three times with cold PBS, and incubated with RNase A (0.1 mg/ml, Taraka) diluted in prechilled PBS. Then, PI (propidium iodide, 20 mg/ml) was added. The cell cycle was measured using FACS Calibur flow cytometer (Franklin Lake, NJ). The data were analyzed by Cell Quest software (Franklin Lake, NJ).

For the detection of cell apoptosis, all samples were washed in phosphate-buffered saline and resuspended in 200 *μ*l binding buffer. Next, 5 *μ*l Annexin-V-fluorescein isothiocyanate, and 10 *μ*l propidium iodide (PI; 1 *μ*g/ml) was added, and the cell suspenson was incubated in dark room for 1 h at room temperature. Then, FACS Calibur flow cytometer (BD Biosciences, Franklin Lakes, NJ, USA) was used for measurement, and CellQuest software (BD Biosciences) was used for data analysis.

### 2.10. Western Blot Analysis

Western blot analysis was performed according to the manufacturer's instructions. The antibodies used in this study were shown as follows: HMGA1 (1 : 1000, Abcam, ab4078), AKT (1 : 5000, Abcam, ab179463), PI3K (1 : 1000, BIOSS, bsm-33219 M), p-AKT (1 : 5000, Abcam, ab81283), p-PI3K (1 : 2000, Affinity, AF3242), MMP9 (1 : 2500, Proteintech, 10375-2-AP), and GAPDH (1 : 6000, Abcam, ab8245).

### 2.11. Real-Time PCR

Total RNA was extracted with Trizol reagent (Takara, Japan), and then ReverTra Ace qPCR RT kit (Toyobo, Japan) was used for reverse transcription. Real-time PCR was performed on a CFX Connect real-time system (Biorad, USA). The conditions were as follows: initial denaturation (95°C for 5 min) and then 40 cycles of three-step PCR (95°C for 40 s, 60°C for 50 s, and 72°C for 30 s). Invitrogen designed and provided all primers.

The primer sequences were as follows: HMGA1, sense 5′-GCTGGTAGGGAGTCAGAAGGA-3′, and antisense 5′-TGGTGGTTTTCCGGGTCTTG-3′; GAPDH, sense 5′-TGCACCACCAACTGCTTAGC-3′, and antisense 5′-GGCATGGACTGTGGTCATGAG-3′.

### 2.12. Immunohistochemistry (IHC)

For immunohistochemical analysis, 39 breast cancer tissues and paired adjacent noncancer samples were included. The clinical characteristics are shown in Table 1. Immunohistochemistry was carried out according to the manufacturer's instructions (Boster, Wuhan, China). HMGA1 antibody was purchased from Abcam (1 : 200, ab4078). The results were evaluated as described previously [14].

### 2.13. Statistical Analysis

SPSS 20.0 was applied for statistical analysis. Data were presented as mean ± SD. Statistical comparisons between groups were analyzed using Student's *t*-test. *P* < 0.05 indicated statistical significance.

## 3. Result

### 3.1. HMGA1 Expression Is Elevated in Breast Cancer Tissues

**Figure 1:**
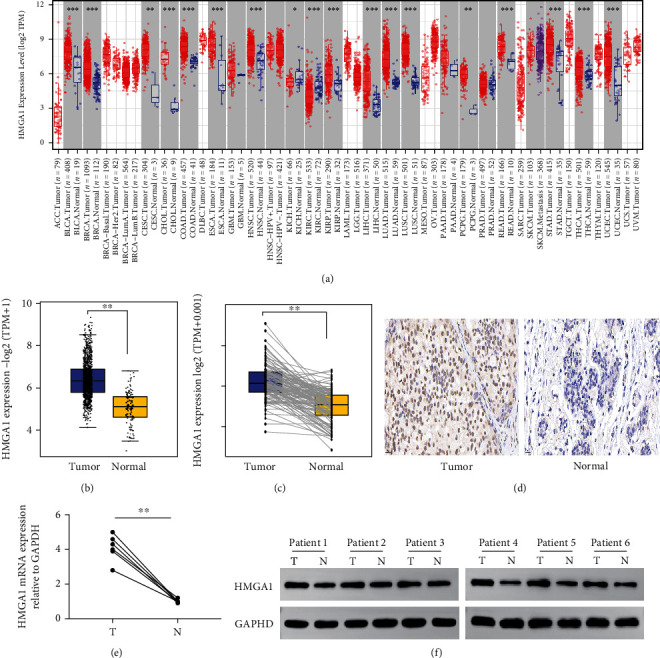
HMGA1 expression analysis in breast cancer. (a) HMGA1 expression in tumor and normal tissues in TIMER database. (b) HMGA1 expression in tumor and normal tissues from TCGA. (c) HMGA1 expression in paired tumor and normal tissues from TCGA. Data were shown as mean ± SD. (d) HMGA1 protein expression in breast cancer and paired adjacent noncancer tissues. (e) Relative mRNA level of HMGA1 to GAPDH in six-paired breast carcinoma (t) and their adjacent nontumor tissues (N). (f) Detection of HMGA1 protein levels by western blotting in six-paired breast carcinoma (T) and their adjacent nontumor tissues (N). (^∗^*P* < 0.05, ^∗∗^*P* < 0.01, ^∗∗∗^*P* < 0.001, and ^∗∗∗∗^*P* < 0.0001).

In analysis of RNA-seq data in TIMER database, we found that the expression of HMGA1 in most cancer tissues was higher than that in normal tissues, including breast and other 17 types of cancers (Figure 1(a)). Additionally, high HMGA1 expression was observed in breast cancer in the TCGA cohort compared to normal breast tissues (Figure 1(b)). Moreover, the level of HMGA1 was prominently increased in breast cancer tissues compared with the adjacent nontumor tissues (Figure 1(c)).

In view of the clinical characteristics of HMGA1 mRNA expression in breast cancer, we try to evaluate the HMGA1 protein expression in breast cancer tissues and paired adjacent nontumor tissues by immunohistochemical staining (IHC). As shown in Figure 1(d), HMGA1 protein positive staining is located in the nucleus and is upregulated in breast cancer tissue.

Next, we examined the expression of HMGA1 mRNA and protein in six breast cancer tissues and paired adjacent nontumor tissues and got consistent finding in TCGA database. The qRT-PCR and western blot results showed higher HMGA1 expression in cancer tissues comparing with that in normal adjacent tissues (Figures 1(e) and 1(f)). These data suggest that HMGA1 may play an important role in the pathogenesis of breast cancer.

### 3.2. High mRNA Level of HMGA1 Predicts Poor Survival of Breast Cancer Patients

**Figure 2:**
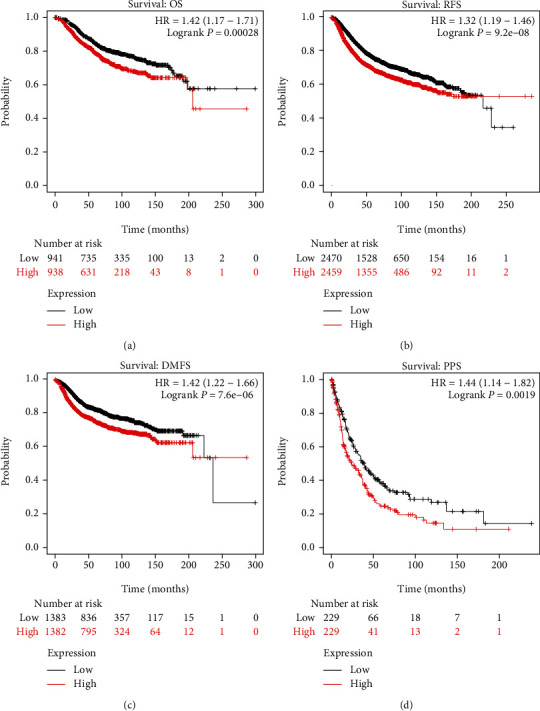
High expression of HMGA1 predicted the worse outcome of breast cancer patients. Determination of prognostic value of HMGA1 mRNA expression in KM plotter database. Affymetrix ID is valid: 206074_s_at (HMGA1). Survival curves were plotted for all breast cancer patients with different survival events. (a) Survival: overall survival (OS), *n* = 1,879. (b) Survival: relapse-free survival (RFS), *n* = 4,929. (c) Survival: distance metastasis-free survival (DMFS), *n* = 2,765. (e) Survival: postprogression survival (PPS), *n* = 458.

To systematically evaluate the association between HMGA1 expression and patient survival in breast cancer, we performed the survival curves from Kaplan–Meier plotter and bc-GenExMiner online database. We found higher HMGA1 expression predict poor prognosis: the OS (Figure 2(a), HR = 1.42, 95% CI = 1.17 to 1.71, *P* = 0.00028), RFS (Figure 2(b), HR = 1.32, 95% CI = 1.19 to 1.46, *P* = 9.2*e* − 08), DMFS (Figure 2(c), HR = 1.42, 95% CI = 1.22 to 1.66, *P* = 7.6*e* − 06), and PPS (Figure 2(d), HR = 1.44, 95% CI = 1.14 to 1.82, *P* = 0.0019). Additionally, consistent results were obtained from bc-GenExMiner (Supplementary Figure 2). These finding indicate that HMGA1 is a potential oncogene in breast cancer.

### 3.3. Association between HMGA1 Expression and Clinical Characteristics in Breast Cancer

**Figure 3:**
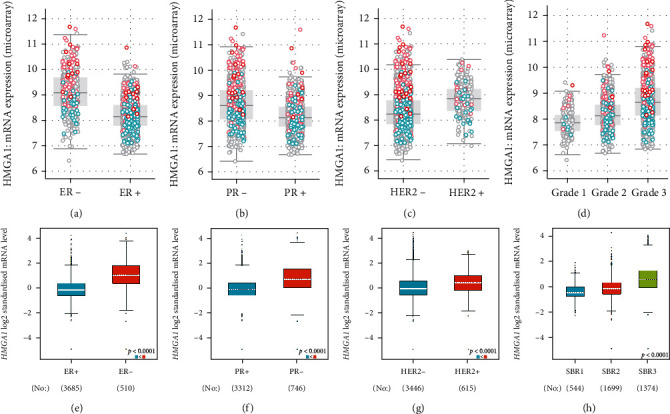
The clinical significance of HMGA1 in breast cancer. (a–d) The data of HMGA1 was obtained from breast cancer (METABRIC, Nature 2012 and Nat Commun 2016) in the cBioPortal for Cancer Genomics (http://www.cbioportal.org). The correlation between the mRNA expression of HMGA1 and (a) ER status, (b) PR status, (c) HER2 status, and (d) neoplasm histologic grade of breast cancer. (e–h) The data of HMGA1 was obtained from bc-GenExMiner (http://bcgenex.centregauduheau.fr/). The association between HMGA1 mRNA level (Log2) and (e) ER status, (f) PR status, (g) HER2 status, and (h) Scarff Bloom and Richardson grade (SBR).

The association between HMGA1 mRNA expression and the clinical characteristics was investigated using mRNA expression *z* score (U133 microarray only) data available from cBioportal, and the genome profiles of 2,509 BC patients were analyzed. As shown in Figure 3, HMGA1 expression was significantly correlated with ER (Figure 3(a)), PR (Figure 3(b)), HER2 (Figure 3(c)), and grade (Figure 3(d)) in BC patients. We also used bc-GenExMiner which contained molecular subtyping of 4,384 breast cancers to analyze the relationship between HMGA1 expression and clinical characteristics. As expected, the consistent results were gotten from bc-GenExMiner (Figures 3(e)–3(h)). Additionally, high HMGA1 was significantly related with lymph node metastasis and Nottingham Prognostic Index (NPI). It was worth mentioning that HMGA1 expression was obviously lower in luminal A BC than in the other subtypes in cohort from both bc-GenExMiner and cBioportal, and it was obviously higher in basal-like breast cancer than in the other subtypes (Supplementary Figure 3).

### 3.4. Correlation and Enrichment Analyses

**Figure 4:**
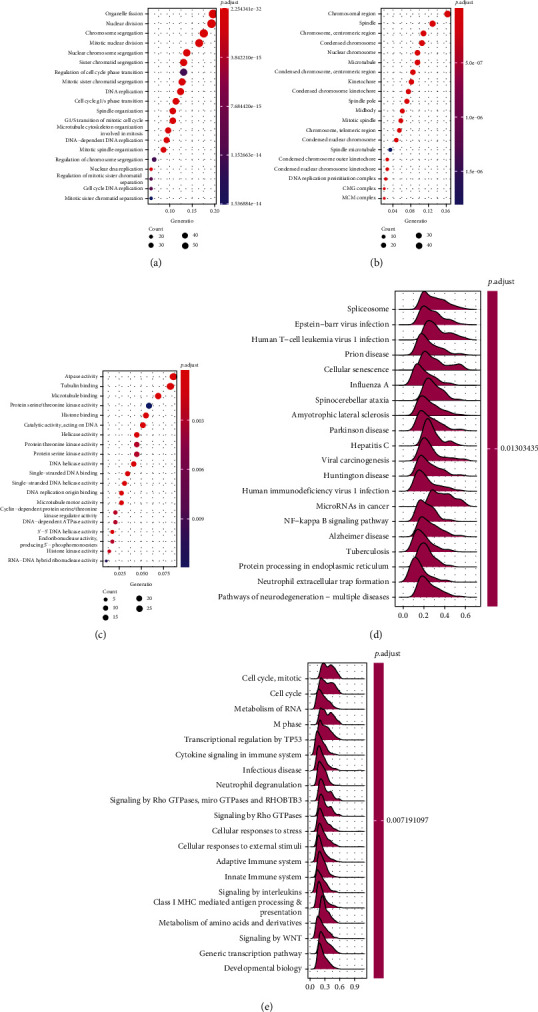
Enrichment analysis of HMGA1 in breast cancer. (a–c) Significant Gene Ontology terms of top 300 genes most positively associated with HMGA1, including (a) biological processes, (b) cell component, and (c) molecular function. (d, e) Significant gene set enrichment analysis (GSEA) results of HMGA1, including (d) KEGG pathways and (e) Reactome pathways.

To further explore the potential molecular function of HMGA1 in breast carcinogenesis, we used cluster profile GO and KEGG analyses to predict HMGA1-related signaling pathways. GO analysis revealed that HMGA1 was associated with cell cycle transition, DNA replication, chromosomal region, protein serine/threonine kinase activity, and ATPase activity (Figures 4(a)–4(c)). According to GSEA analysis, we found that cell cycle, cellular senescence, NF-kappa B signaling pathway, transcriptional regulation by TP53, and signaling by WNT were significantly enriched in samples with high HMGA1 expression (Figures 4(d) and 4(e)).

### 3.5. Knockdown of HMGA1 Inhibits Breast Cancer Cells Growth and Metastasis In Vitro

**Figure 5:**
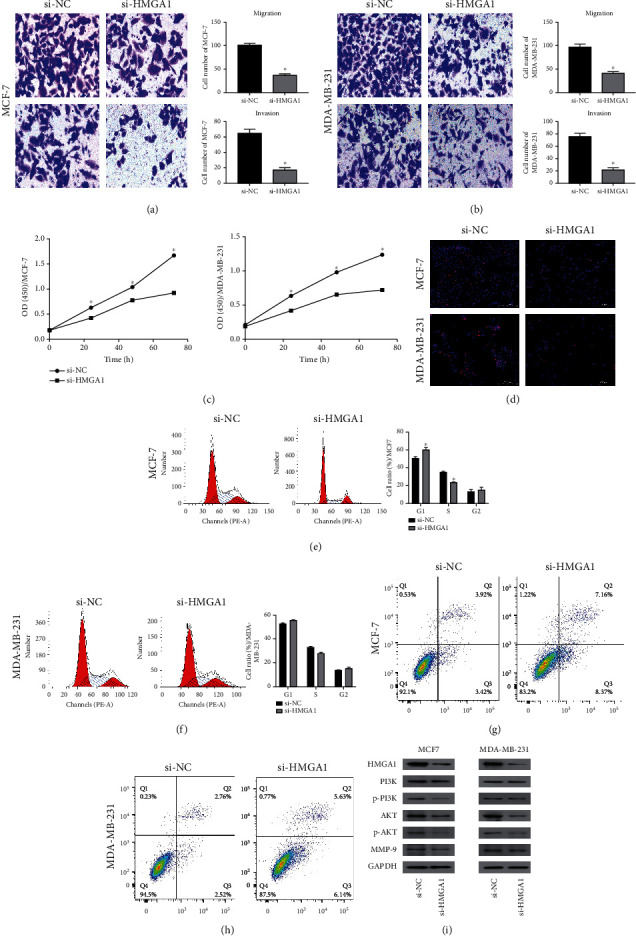
Knockdown of HMGA1 inhibited the proliferation, apoptosis, migration, and invasion of breast cancer cells. (a) The migration and invasion of MCF-7 was examined by transwell assay. (b) The migration and invasion of MDA-MB-231 was examined by transwell assay. (c) The proliferation of MCF-7/MDA-MB-231 cells was examined by CCK-8 assay. (d) The proliferation of MCF-7/MDA-MB-231 cells was examined by EdU assay. (e) Representative cell cycle images of MCF-7 at 48 h after transfection with 100 nmol siNC or 100 nmol specific siHMGA1 by flow cytometry. (f) Representative cell cycle images of MDA-MB-231 at 48 h after transfection with 100 nmol siNC or 100 nmol specific siHMGA1 by flow cytometry. (g) Representative FACS analyses for the induction of apoptosis in HMGA1 on MCF-7 and MCF-7/si-HMGA1 cells. (h) Representative FACS analyses for the induction of apoptosis in HMGA1 on MDA-MB-231, and MDA-MB-231/si-HMGA1 cells. (i) The expression levels of indicated proteins were evaluated by western blot in control and HMGA1-silenced MCF-7/MDA-MB-231cells. Data were shown as mean ± SD. (^∗^*P* < 0.05).

To investigate the biological function of HMGA1 in breast cancer, we transfected MCF-7 and MDA-MB-231 cells with siRNA selectively targeting HMGA1. The transwell migration/invasion assay showed that HMGA1 knockdown significantly inhibited MCF-7/MDA-MB-231 cell migration and invasion (Figures 5(a) and 5(b)). Besides, CCK8 and EdU analysis demonstrated that silencing HMGA1 inhibited the proliferative capacity of MCF-7 cells and MDA-MB-231 cells (Figures 5(c) and 5(d)). Furthermore, HMGA1 silencing in MCF-7 cells resulted in an increase in the number of cells in the G1 phase from 50.91 ± 1.44% to 60.42 ± 2.28% and a decrease in the number of cells in the S phase from 35.4 ± 0.74% to 23.87 ± 0.4% (Figure 5(e)). G1 phase arrest (from 53.16 ± 0.51% to 56.09 ± 0.32%) and S phase inhibition (from 33.13 ± 0.76% to 28.31 ± 0.62%) were also found in MDA-MB-231 cells after silencing HMGA1 expression (Figure 5(f)). Apoptosis of breast cancer cells was detected by Annexin-V and PI double-staining and measured by flow cytometry. As shown in Figures 5(g) and 5(h), the apoptotic rate of MCF-7/si HMGA1 (15.53%) was significantly higher than MCF-7 (7.34%). In addition, HMGA1 silencing resulted in 11.77% of MDA-MB-231 cells being apoptotic, while the apoptotic rate of MDA-MB-231 was 5.28% (*P* < 0.05). These results suggest that HMGA1 may be involved in breast cancer progression and metastasis.

PI3K/AKT pathway has been reported playing a vital role for tumor initiation and progression in many cancers, such as uveal melanoma, pancreatic adenocarcinoma, and hepatocellular carcinoma [15–17]. Therefore, we further assessed the impact of HMGA1 on PI3K/AKT/MMP-9 pathway in breast cancer cells. Downregulation of HMGA1 expression was observed in MCF-7 cells and MDA-MB-231 cells transfected with HMGA1 siRNA by western blot. Subsequently, we find significant decrease in the expression of PI3K, p-PI3K, AKT, p-Akt, and MMP-9 in MCF-7/MDA-MB-231 cells transfected with si-HMGA1 (all *P* < 0.05) (Figure 5(i)). Therefore, our data show that HMGA1 is a regulator of the PI3K/ATK/MMP-9 pathway, as predicted by our GO analysis.

## 4. Discussion

In recent years, HMGA1 was reported to play regulatory roles in various diseases. For example, it was reported that the expression of HMGA1 was upregulated in hepatocellular carcinoma and related to prognosis [18]. Downregulation of HMGA1 expression inhibited the proliferation, migration, and invasion of thyroid cancer cells [19]. Similarly, we had reported that HMGA1 exacerbates tumor growth and accelerates migration/invasion of cervical cancer [11], suggesting that HMGA1 might be a target oncoprotein. However, the expression pattern of HMGA1 in most human tumors and its prognostic values remain unclear. Qi et al. reported that HMGA1 expression was closely associated with the clinical stage and histological grade of breast cancer in 169 breast cancer tissues and 37 normal breast tissues [20]. Gorbounov's article mentioned that high expression of HMGA1 in breast cancer predicted poor overall survival [21]. However, the specific function and mechanism of HMGA1 still need further exploration in breast cancer. Here, we analyzed the data from online datasets to compare the expression of HMGA1, and found higher expression was observed in 1085 tumor tissues when compared with 112 normal tissues, indicating that it might be a novel oncogene. HMGA1 expression was obviously high in breast cancer compared with normal tissue and the adjacent tissues according to TCGA data. Furthermore, we verified the above conclusions by immunohistochemistry.

The KM plotter was used in our study, which can evaluate the correlation between gene expression and survival in samples from 21 tumor types including breast cancer [22]. Affymetrix ID is valid: 206074_s_at (HMGA1). The survival curves showed that, for thousands of breast cancer patients, higher HMGA1 mRNA levels were associated with worse OS, worse RFS, worse DMFS, and worse PPS. To systematically test the association between HMGA1 expression and specifical receptors, 2509 breast cancers in “cBioportal” and 4384 breast cancers in “bc-GenExMiner” were analyzed, respectively, and got the consistent conclusions; high HMGA1 expression was found in ER-negative group, PR-negative group, and HER2-positive group and was significantly related with high-grade pathological group and lymph node metastasis. NPI is a clinicopathological classification system based on tumor size, histological grade, and lymph node status, and our study also showed a positive association between HMGA1 expression with NPI. Moreover, HMGA1 was obviously higher in basal-like breast cancer than in the other subtypes, which dictated therapeutic guidance and portended prognosis.

In addition, we utilized functional and pathway enrichment analysis to explore the potential mechanisms of HMGA1 in breast cancer. The results showed that high expression of HMGA1 was associated with cell cycle transition, DNA replication, chromosomal region, protein serine/threonine kinase activity, and ATPase activity in breast cancer. HMGA1 has previously been reported to be related to malignant cellular behavior in human cancers [23–25]. Although extensive correlative evidences indicated that HMGA1 play a role in tumor metastasis, few studies have shown the direct functional relationship between HMGA1 expression and invasion/metastasis in breast cancer. By gene silencing experiments, we found that HMGA1 downregulation could inhibit cell proliferation through inducing G1 phase arrest and S phase inhibition, induce cell apoptosis, and weaken the migrating and invasive ability of breast cancer cells.

PI3K is a key downstream signal of growth factor tyrosine kinases, participating in the recruitment and activation of multiple cellular targets including AKT [26]. In turn, AKT activation helps regulating cell growth, cell survival, and cell mobility [27]. Besides, accumulating studies suggest that MMPs could involve in the early stage of cancer metastasis [28–30]. PI3K/AKT/MMP-9 pathway has been gradually recognized to play an indispensable role in cell growth, proliferation, and differentiation [31–33]. Previous studies have reported that the PI3K/AKT/MMP-9 pathway is a unique downstream pathway of HMGA1, which is critical for uveal melanoma cell proliferation, survival, and migration [32]. Our study showed that the downregulation of HMGA1 inhibits proliferation, invasion and metastasis, and induce apoptosis of breast cancer cells and provides evidence that HMGA1 expression mediates cellular malignant biological behavior through PI3K/Akt/MMP-9 dependent pathway, which is consistent with previous findings in uveal melanoma. The existing findings indicate a key role of HMGA1 in regulating tumor metastasis and predicting prognosis. However, the detailed mechanism of HMGA1 in breast cancer still needs further exploration.

In summary, our results showed that the HMGA1 level was significantly upregulated in breast cancer tissues and closely related to clinical feature. Higher HMGA1 predicted poor survival in breast cancer patients. Downregulation of HMGA1 induces apoptosis and cell cycle arrest and inhibits cell proliferation, migration, and invasion in breast cancer. Our findings indicate that HMGA1 may be a critical prognostic indicator and potential therapeutic target of breast cancer.

## Figures and Tables

**Table 1:** The clinicopathological parameters of cases used in IHC.

	No.
Age	
(i) ≥ 50	22
(ii) <50	17
Tumor subtype	
(i) Normal breast-like	4
(ii) Luminal A	10
(iii) Luminal B	14
(iv) HER2	7
(v) Basal-like	4
Lymph node invasion	
Negative	20
(i) Positive	19
Histological grade	
(ii) I	14
(iii) II	13
(iv) III	12

## Data Availability

The data used to support the findings of this study are included within the article and the ementary information files.

## Supplementary Materials

Supplementary 1 Supplementary Figure 1: screening and detecting the effect of siRNA in breast cancer cells.

Supplementary 2 Supplementary Figure 2: prognostic value of HMGA1 mRNA expression in bc-GenExMiner database.

Supplementary 3 Supplementary Figure 3: association between HMGA1 expression and lymph node metastasis, NPI (Nottingham Prognostic Index) stage, and breast cancer subtypes.
