# Supplementary figures and images for: HMGA1 As a Potential Prognostic and Therapeutic Biomarker in Breast Cancer
Source: Dis Markers. 2022 Nov 26;2022:7466555. doi: 10.1155/2022/7466555 (PMC9720233; doi:10.1155/2022/7466555)

**A****MCF-7**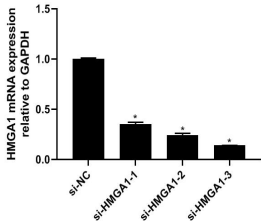**B****MDA-MB-231**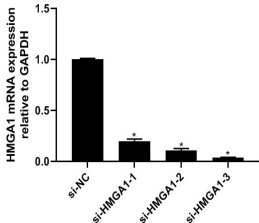

Supplement: Supplementary 1 — Supplementary Figure 1: screening and detecting the effect of siRNA in breast cancer cells. [file 7466555.f1.pdf]

A

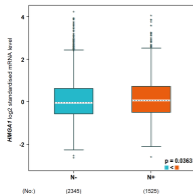

B

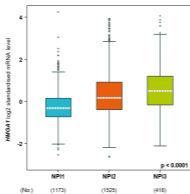

C

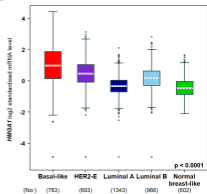

D

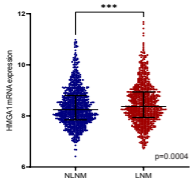

E

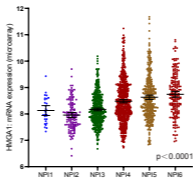

F

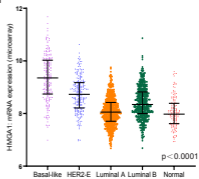

Supplement: Supplementary 3 — Supplementary Figure 3: association between HMGA1 expression and lymph node metastasis, NPI (Nottingham Prognostic Index) stage, and breast cancer subtypes. [file 7466555.f3.pdf]
