# Supplementary material for: HMGA1 As a Potential Prognostic and Therapeutic Biomarker in Breast Cancer
Source: Dis Markers. 2022 Nov 26;2022:7466555. doi: 10.1155/2022/7466555 (PMC9720233; doi:10.1155/2022/7466555)
Supplement: Supplementary 2 — Supplementary Figure 2: prognostic value of HMGA1 mRNA expression in bc-GenExMiner database. [file 7466555.f2.pdf]

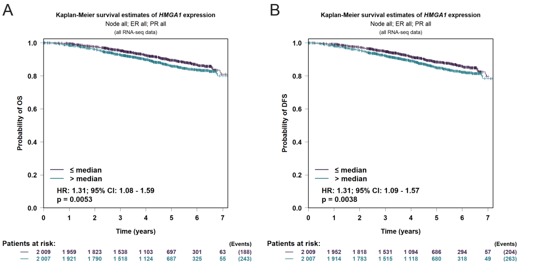
A
Kaplan-Meier survival estimates of HMGA1 expression
Node all; ER all; PR all
(all RNA-seq data)
Probability of OS
Time (years)
≤ median
> median
HR: 1.31; 95% CI: 1.08 - 1.59
p = 0.0053
Patients at risk:
(Events)
2 009 1 959 1 823 1 538 1 103 697 301 63 (188)
2 007 1 921 1 790 1 518 1 124 687 325 55 (243)
B
Kaplan-Meier survival estimates of HMGA1 expression
Node all; ER all; PR all
(all RNA-seq data)
Probability of DFS
Time (years)
≤ median
> median
HR: 1.31; 95% CI: 1.09 - 1.57
p = 0.0038
Patients at risk:
(Events)
2 009 1 952 1 818 1 531 1 094 686 294 57 (204)
2 007 1 914 1 783 1 515 1 118 680 318 49 (263)
